# GNSS total variometric approach: first demonstration of a tool for real-time tsunami genesis estimation

**DOI:** 10.1038/s41598-021-82532-6

**Published:** 2021-02-04

**Authors:** Michela Ravanelli, Giovanni Occhipinti, Giorgio Savastano, Attila Komjathy, Esayas B. Shume, Mattia Crespi

**Affiliations:** 1 Sapienza University of Rome, via Eudossiana 18, Rome, 00184 Italy; 2 Université de Paris, Institut de Physique du Globe de Paris, CNRS, 75005 Paris, France; 3 Institut Universitaire de France, Paris, France; 4 Jet Propulsion Laboratory, 4800 Oak Grove Dr, Pasadena, CA 91109 USA; 5 Present Address: Spire Global, Inc., 33 Rue Sainte-Zithe, 2763 Luxembourg, Luxembourg; 6 Present Address: California Institute of Technology, 1200 E California Blvd, Pasadena, CA 91125 USA

**Keywords:** Geophysics, Natural hazards

## Abstract

Global Navigation Satellite System (GNSS) is used in seismology to study the ground displacements as well as to monitor the ionospheric total electron content (TEC) perturbations following seismic events. The aim of this work is to combine these two observations in one real-time method based on the Total Variometric Approach (TVA) to include the GNSS real-time data stream in future warning systems and tsunami genesis estimation observing both, ground motion and TEC. Our TVA couples together the Variometric Approach for Displacement Analysis Stand-alone Engine (VADASE) with the Variometric Approach for Real-Time Ionosphere Observation (VARION) algorithms. We apply the TVA to the Mw 8.3 Illapel earthquake, that occurred in Chile on September 16, 2015, and we demonstrate the coherence of the earthquake ground shaking and the TEC perturbation by using the same GNSS data stream in a real-time scenario. Nominally, we also highlight a stronger kinetic energy released in the north of the epicenter and visible in both, the ground motion and the TEC perturbation detect at 30 s and around 9.5 min after the rupture respectively. The high spatial resolution of ionospheric TEC measurement seems to match with the extent of the seismic source. The GNSS data stream by TVA of both the ground and ionospheric measurement opens today new perspectives to real-time warning systems for tsunami genesis estimation.

## Introduction

Natural hazards such as earthquakes and the subsequent tsunamis generate atmospheric acoustic-gravity waves that propagate upward to the ionosphere where they may be detectable by ionospheric sounding techniques^1^. In particular, at the epicentral area, the uplift at the surface—produced by the seismic rupture—induces, by dynamic coupling with the atmosphere, an acoustic-gravity wave (AGW$_{epi}$). During the upward propagation the AGW$_{epi}$ is strongly amplified by the double effects of the decrease of the air density $\rho$ and the conservation of the kinetic energy $\rho v^2$, under the adiabatic hypothesis of the atmosphere. Consequently the oscillation *v* induced by the crossing of the AGW$_{epi}$ is becoming large enough to generate strong perturbations in the ionospheric plasma, mainly where its density is near the maximum (F-layer, altitude about 300 km)^2^.

Different techniques have been already applied to detect the ionospheric post-seismic perturbations: ionosondes^3^, HF Doppler sounders^4,5^, airglow cameras^1,6–8^, over-the-horizon radar^8,9^ and GNSS sTEC (slant TEC, TEC on the satellite-receiver line-of-sight LoS) observations^10–14^. This last technique received more and more attention due to the wide and continuously increasing worldwide availability of GNSS permanent stations. GNSS sTEC became a major tool to study the ionosphere^15^, which can be affected by geophysical events such as earthquake^1,2,16^, tsunami^17–20^, volcano explosions^21–23^; and man-made events such as explosions^24,25^ and rocket launches^26–28^. Recently, an algorithm was developed^29,30^, named VARION (Variometric Approach for Real-Time Ionosphere Observation), able to estimate sTEC variations from GNSS observations in real time. GNSS-sTEC measurements are commonly referred to the altitude of the F-layer along the LoS, called the ionospheric piercing point (IPP). The AGW$_{epi}$ for large events (Mw > 7) is systematically detectable near the epicenter^2^, within 1000 km, and they move vertically at the speed of sound c$_s$. In the Earth atmosphere c$_s$ varies with the altitude (from several hundreds m/s—near sea level—to about 1 km/s at 400 km of altitude); c$_s$ ranges from 800 to 1000 m/s at the height of the ionospheric F-layer ( 300 km), where the electron density reaches the maximum^11^. Overall, it takes around 8 min for the AGW$_{epi}$ to be detectable by GNSS-sTEC observations.

The recent work of Manta *et al.*^16^ clearly shows the empirical relationship between the TEC perturbations and the maximum volume of the displaced water during the tsunami genesis (source extent times the max uplift at the source). Authors highlighted the potential use of GNSS-sTEC observations for tsunami genesis estimation at 8 min after the rupture to support conventional tsunami warning systems. They introduced the ionospheric tsunami power index (ITPI) empirically related to the maximum volume of displaced water (in km^3^) during the tsunami genesis to discriminate tsunamigenic earthquakes.

On the other hand, it has been known and tested that earthquakes induced ground shaking and coseismic displacements that can be effectively recorded using GNSS as well^31–36^. The ground motion measured by GNSS is benefiting of its relevant feature not to be affected by the saturation problem, which can impact seismometers located near the epicenters of strong earthquakes. A great effort in the last decade has been done in order to explore the contribution of GNSS data in the future tsunami warning systems in order to improve their reliability of the responses. Ground motion measured by GNSS receivers has been used to estimate the magnitude^37^, moment tensor^38^ as well as finite source^39^, in particular for tsunamigenic events and to improve the tsunami genesis estimation^40^. Also in this case, the real-time approach, named VADASE (Variometric Approach for Displacements Analysis Stand-Alond Engine), was proposed by Colosimo *et al.*^41^ and refined in Fratarcangeli *et al.*^42^ to estimate seismic waveforms and coseismic displacements.

We introduce here the dual GNSS real-time contribution using the same data-stream from permanent stations for both ground shaking and TEC estimation, to explore the potential improvement of tsunami genesis estimation. We coupled the VADASE and VARION algorithms—the former being able to estimate surface waveforms and co seismic displacements^41,42^, and the latter is able to compute sTEC perturbations in real-time^29,30^.

The final algorithm, named Total Variometric Approach (TVA), was applied to the moderate tsunamigenic earthquake of Illapel (Chile, September 16, 2015, Mw 8.3). We analyse the GNSS permanent stations already providing real-time data stream; as well as the GNSS stations (not real-time) but used here in a real-time scenario to improve the coverage of our network. We also support the GNSS ground motion observations with traditional accelerometer measurements.

In particular, we first introduce the Illapel event and the response of tsunami warning systems; then we detail the dataset and the methodology used in the investigation; and we finally present our results and conclusions.

## Earthquake information and dataset

The 2015 Illapel earthquake, in Chile, occurred 46 km offshore of the Coquimbo region on September 16 at 19:54:33 Chile Standard Time (22:54:33 UTC), with a moment magnitude of Mw 8.3. The initial quake lasted between 3 and 5 min and it was followed by several aftershocks greater than moment magnitude 6.0, and two of them exceeded 7.0^43^. The earthquake occurred on thrust faults along the boundary of the Nazca and South American plates. The region frequently produces large earthquakes that induce deadly tsunamis.

A tsunami threat message from NOAA Pacific Tsunami Warning Center was issued 7 min after the main shock^44,45^ and Servicio Hidrográfico y Oceanográfico de la Armada (the organization in charge of the Chile’s National Tsunami Warning System) issued the first tsunami alarm 8 min^46^ after the earthquake: despite the prompt evacuation, eight causalities were attributed to the tsunami^47^. The first tsunami waves arrived on the Chilean coast within 10 min^48^, with a series of waves reaching at least 4.5 m^49^ on the closer coasts and produced severe damages and losses.

**Figure 1:**
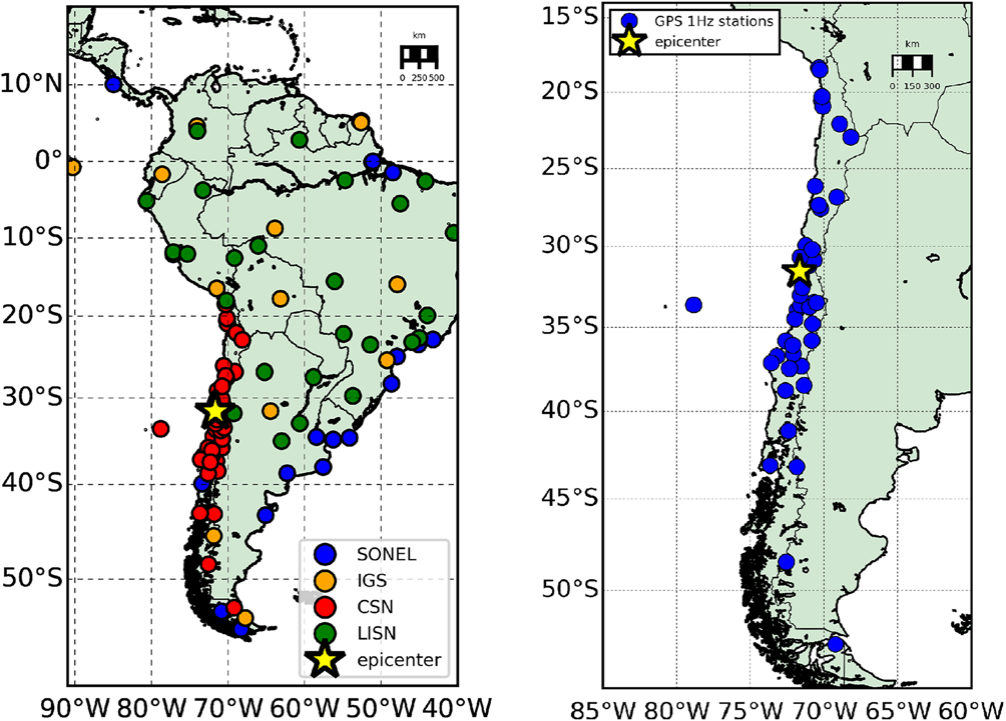
Left—Map with the epicenter of the earthquake and the 118 GPS permanent stations at low-rate data sampling (10, 15, 30 s) used in VARION processing for sTEC variation estimation; GPS stations from CSN, SONEL, IGS and LISN in red, blue, orange and green respectively. Right—Map with the epicenter of the earthquake and the 44 GPS permanent stations at high-rate data sampling (1 s) used in VADASE processing for ground shaking and kinetic energy estimation: CSN stations in blue.

Different datasets were employed for VADASE and VARION algorithm. Nominally, 44 GPS stations at 1 Hz rate from *Centro Sismológico Nacional, Universidad de Chile* were employed for VADASE algorithm: the dataset is shown in the right panel of Fig. 1. The high-rate data (1 Hz or more) are required to avoid aliasing in the estimation of ground shaking and waveforms.

Data from 118 GPS stations, mostly located in Chile but also spread all over the South-American continent, were processed with VARION, as it is shown in Fig. 1 (left panel). Nominally, 48 GPS stations (including the previous 44, since they also supply data at 15 s rate), located in Chile were kindly provided by the *Centro Sismológico Nacional, Universidad de Chile* (CSN). Other observations were gathered from 15 GPS stations of *International GNSS service* (IGS), from 26 GPS stations of *Système d’Observation du Niveau des Eaux Littorales* (SONEL) network and from 29 GPS stations of *Low-Latitude Ionospheric Sensor Network* (LISN) respectively. These GPS receivers collected data at 10, 15 and 30 s rate.

It is important to underline that only 21 stations of 118 stations can already work in real time. For real time, we mean the possibility for a GNSS receiver to broadcast real-time streaming of GNSS/GPS (RT-GNSS) data in RTCM formats^50^ via the Networked Transport of RTCM via Internet Protocol (NTRIP)^51^. In detail, the GPS stations belonging to CSN are not all able to provide data in real time at this point. Both IGS and SONEL networks include GNSS receivers from national and regional networks: few of them are technically set up to provide data in real time . Nevertheless, the 29 stations from LISN can provide GNSS measurements in near real time (every 15 min). Therefore, it is important to push forward enhancing the GNSS network in a real-time operational approach.

## Ground shaking and coseismic displacements from VADASE

**Figure 2:**
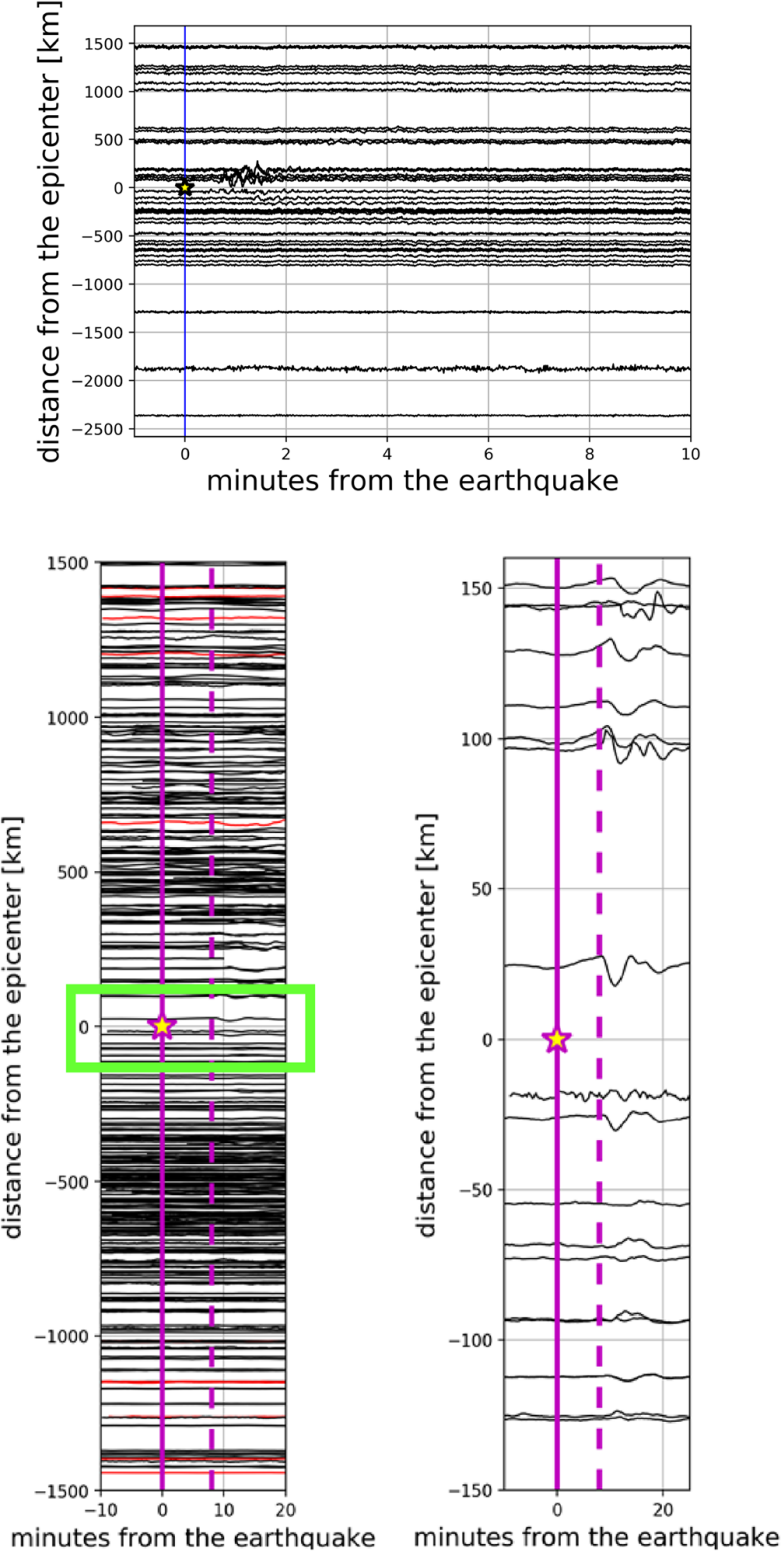
Upper panel—Real-time scenario estimated ground motion (Up component) through VADASE. Bottom panels—sTEC variations for all the satellites in view for all the GPS stations computed through VARION in range of 1500 km from the epicenter (left panel) and a zoom in the range of 150 km (right panel). In both cases, positive and negative distances represent respectively the IPP position at 8 min after the earthquake placed north and south the epicenter.

**Figure 3:**
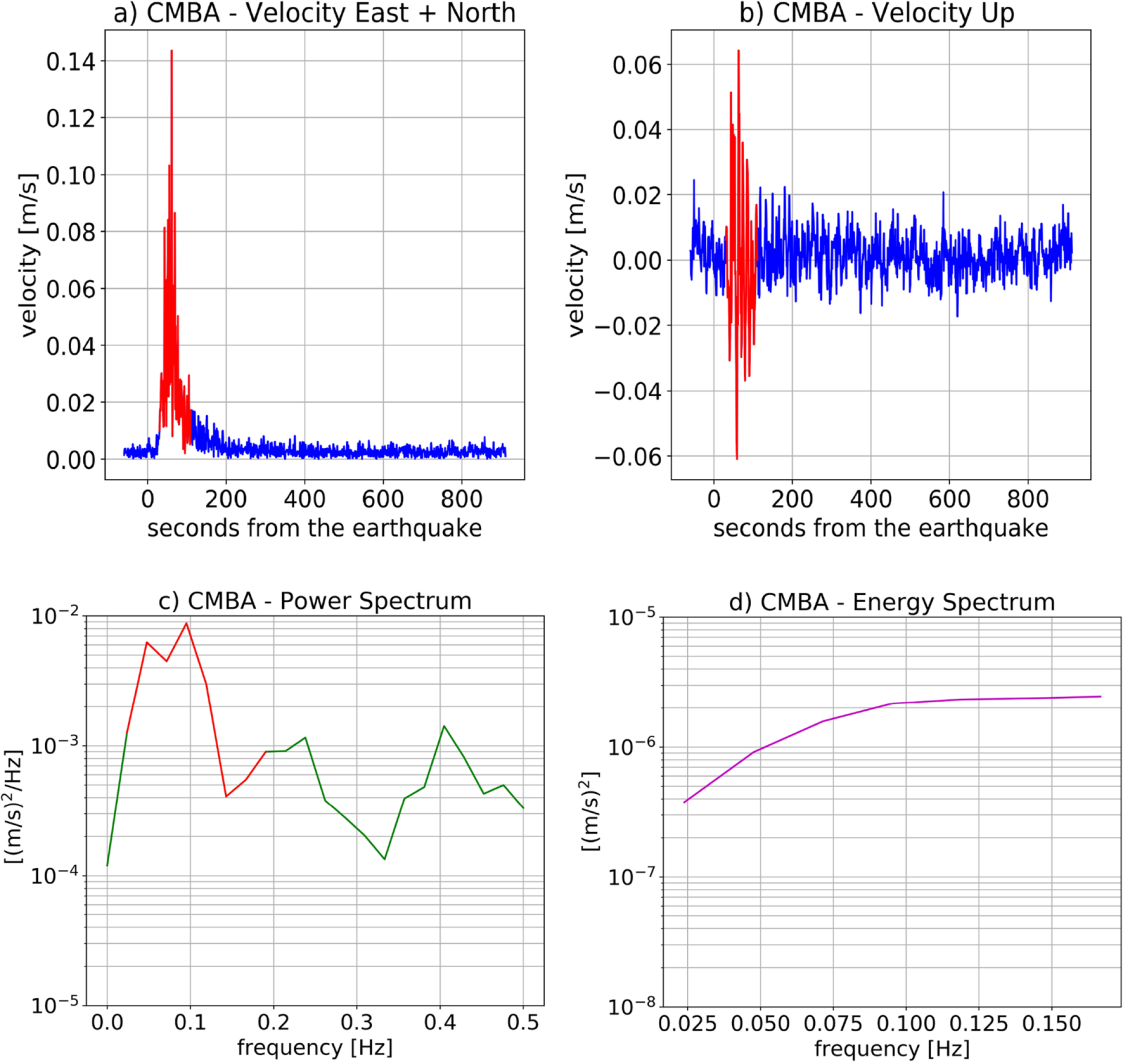
(**a**, **b**) Velocity time series for CMBA GPS station placed north of the epicenter. Specifically, (**a**) and (**b**) show the horizontal component and the Up component of the velocity respectively. The duration was computed on the horizontal component and then applied to the Up component of velocity. The red part highlights the earthquake duration detected with the Levene test. (**c**) The PSD for CMBA station. In red the chosen frequency range (3.3–200 mHz) for the integration. (**d**) The kinetic energy spectrum due to shaking in vertical direction for CMBA station.

GNSS high-rate observations (1 Hz) were used as input for the VADASE algorithm to estimate the east-, north—and up-components of the velocity for each receiver. The upper panel of Fig. 2 show the up-component of velocity: the series are sorted according to GNSS receiver distances (computed with Vincenty’s formula^52^) from the epicenter. Positive and negative distances represent respectively the GNSS stations placed north and south of the epicenter. Velocity time series for the horizontal and the up-components for CMBA station (the closest one to the epicenter towards North) are shown in Fig. 3a,b. The earthquake significant shaking was detected on the basis of the Levene test for equality of variances at around 30 s after the rupture. The moving window was 60 samples, corresponding to 1-min interval, and the significance level for the test was equal to 1%.

The interval corresponding to the earthquake significant shaking represents is selected between 100 and 200 s after the earthquake (highlighted in red in Fig. 3a,b). Nominally, Fig. 3a shows the horizontal component of the velocity for the GNSS stations CMBA, in essence the shaking of the receiver in the horizontal plane. It is important to recall that the shaking duration through Levene test was evaluated on the lesser noisy horizontal component of the velocity (east- and north-components combined), and then the same interval was considered for the up-component.

**Figure 4:**
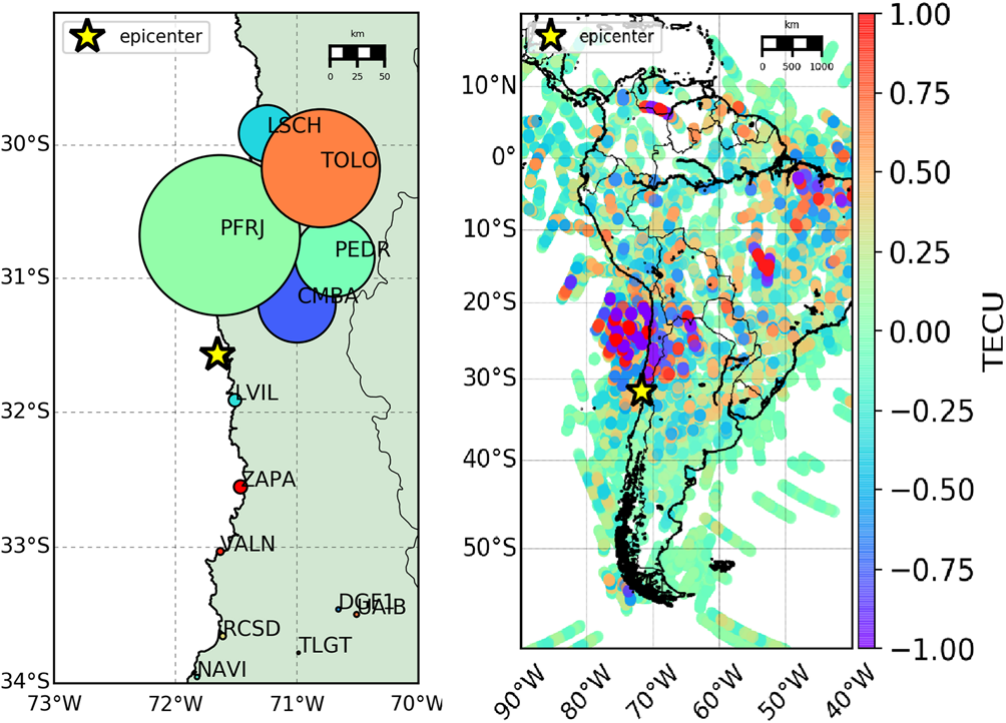
Left panel—Map of the earthquake ground shaking: the dimension of the station markers is proportional to the energy associated to the Up component of the earthquake ground shaking for some stations near the epicenter. It is evident that north placed stations are characterized by a higher energy than south placed ones. Right panel—Space-time sTEC variations at the SIPs for less than two hours (22:54:49-23:59:59 GPS time) on 16$^{th}$ September 2015 for all the satellites in view (cut-off angle set to 20$^\circ$) from all the 118 GPS stations.

The determination of the shaking duration allows to estimate the coseismic displacements and the energy released (see Methods section for computation details) by the earthquake for every GNSS receiver. Left panel of Fig. 4 shows the kinetic energy associated to the Up component for each GNSS station: the size of each station is proportional to the kinetic energy. The kinetic energy (Table 1) released northbound is remarkably higher than the southbound one.

The same procedure was also applied to accelerometer data showing similar results: accelerometers located north are characterized by larger ground shaking than south. In Supplementary Material (SM) we show the accelerometer network (Fig. SM1), the different step to the energy determination for one single accelerometer (Fig. SM2) and the map of released energy by accelerometers (Fig. SM3).

**Figure 5:**
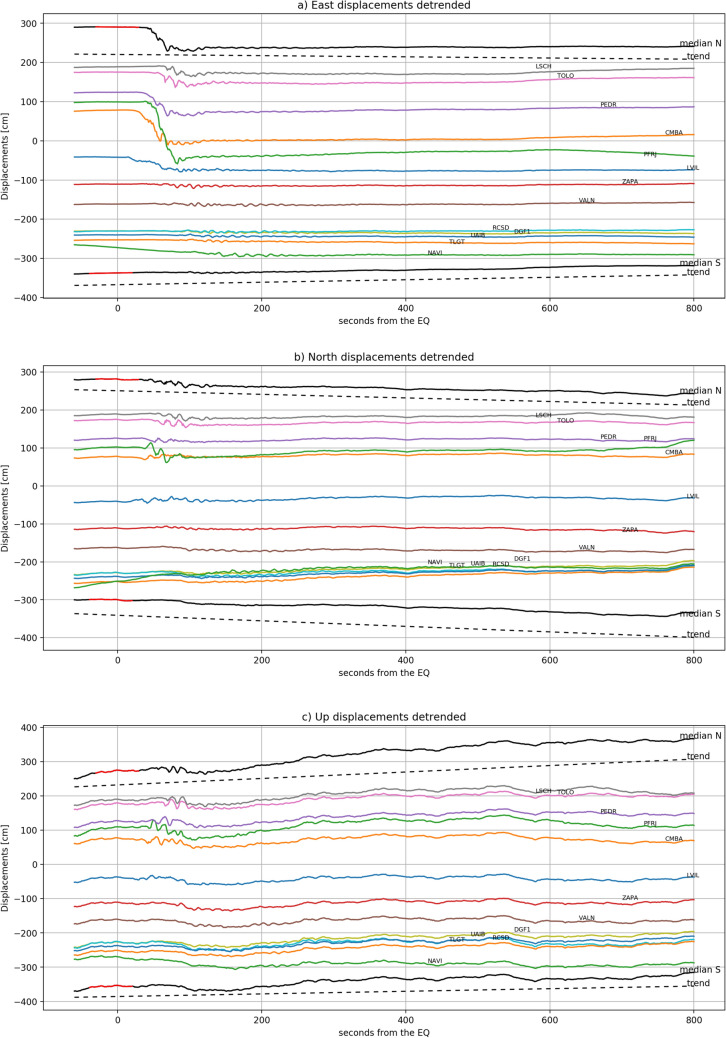
VADASE detrended coseismic displacements for the East (a), North (b) and Up (c) component. The stations are ordered according to their distance from the epicenter (positive distances for north placed stations and negative distances for south placed stations). The spatial median computed for the north and south stations is depicted in black; the red part highlights the interval over which the robust linear fit was estimated, whereas the dashed line represents the extrapolated trend.

In order to visualize the time evolution of the ground displacement, Fig. 5 shows the coseismic de-trended displacements series as computed by integrating the VADASE velocity solutions, the trend is also showed on the same figure (Fig. SM4 shows coseismic displacements before the de-trending operation). Table 1 summarizes the overall coseismic displacements. The largest coseismic displacement occurs for the east-component and this is consistent with the east-northeast movement of the Nazca plate^43^. Moreover, the greatest east displacements take place for the stations placed at north of the epicenter.

We used the shaking duration to determine the energy released by the earthquake in 100–200 s after the earthquake. The Power Spectral Density (PSD) related to this duration was computed from the Up component of the velocities. In Fig. 3c, the PSD respectively for CMBA stations is reported. The normalized kinetic energy associated to the vertical shaking has been calculated by integrating the PSD within the frequency range 3.3-200 mHz. This frequency range was chosen in order to include the acoustic component of the typical AGW$_{epi}$, and this is highlighted in Fig. 3c ; the upper limit of 200 mHz was chosen after a trial and error procedure in order to catch the whole normalized kinetic energy asymmetry. Figure 3d depicts the energy spectrum for CMBA station for the up velocity component.

**Table 1:** Overall coseismic displacements and their standard deviations for the GPS stations within 300 km from the epicenter. The last column shows the value for the energy connected to the Up component of the earthquake ground shaking.

	Stations	Distance	East		North		Up		Energy
			value	$\sigma$	value	$\sigma$	value	$\sigma$	
	–	km	cm	cm	cm	cm	cm	cm	J/kg
North	CMBA	76.91	− 76.76	0.13	0.28	0.62	− 13.56	1.88	2.45 e$^{-6}$
	PFRJ	99.33	− 137.98	0.12	− 19.77	0.57	− 10.36	1.72	5.09 e$^{-6}$
	PEDR	124.01	− 49.50	0.13	− 6.38	0.58	− 1.81	1.75	2.52 e$^{-6}$
	TOLO	176.02	− 27.79	0.12	− 12.63	0.56	− 1.77	1.71	3.74 e$^{-6}$
	LSCH	188.73	− 18.56	0.14	− 10.23	0.64	4.81	1.94	1.80 e$^{-6}$
South	LVIL	40.56	− 33.97	0.11	4.05	1.11	-9.01	1.79	4.02e$^{-7}$
	ZAPA	110.74	− 4.59	0.01	− 1.50	0.95	− 10.04	1.53	4.22 e$^{-7}$
	VALN	161.71	− 1.98	0.15	− 5.14	1.41	2.69	2.28	2.21 e$^{-7}$
	DGF1	229.89	− 4.73	0.10	4.23	0.99	9.97	1.60	1.47 e$^{-7}$
	RCSD	231.23	− 2.16	0.09	− 0.16	0.87	2.41	1.40	1.98 e$^{-7}$

## Ionospheric detection of the AGW$_{epi}$ by VARION

In order to exclude other sources of ionospheric disturbances (e.g., geomagnetic storms), we evaluate relevant geomagnetic indices^53^ during the day of the earthquake. The official planetary K$_p$ index does not reach the value of 4, excluding the possibility of geomagnetic storms on that day^54^. D$_{ST}$ index (Disturbance Storm Time) was about—19 nT before and after the earthquake time^53^. Hence, according to Loewe and Prölss^55^ classification, it is possible to state that no geomagnetic storm was present on that day and so the Illapel earthquake is the main source of the ionospheric perturbations.

**Figure 6:**
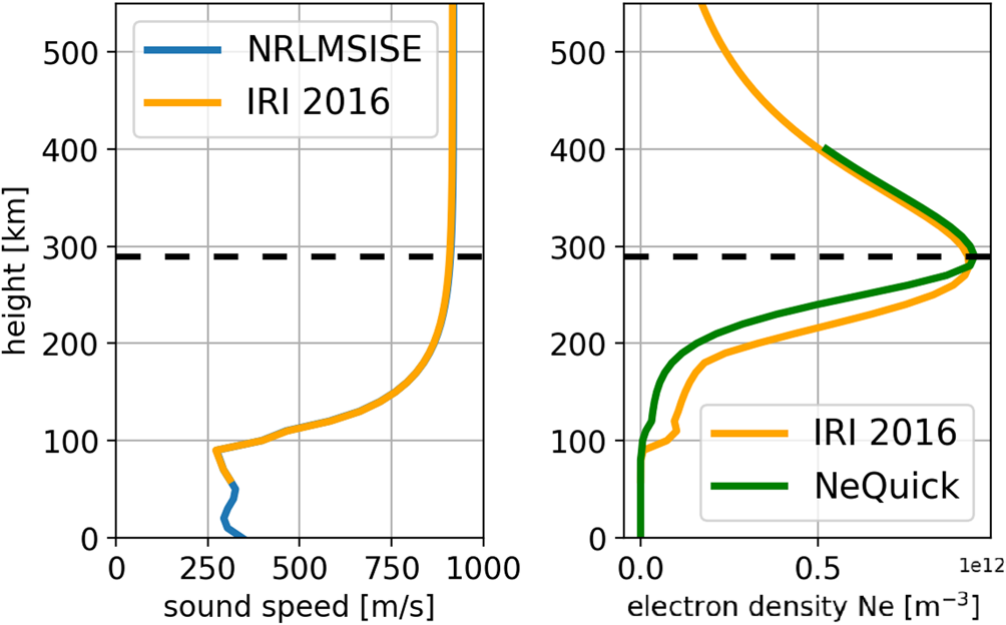
Sound velocity (right) and electron density profile (left) with altitude on September 16$^{th}$ 2015 according to IRI 2016 and Nequick model.

In order to perform the localization of the TEC perturbation detected by the GNSS stations in real-time, we compute the ionospheric piercing points (IPPs) fixing the layer of the ionosphere at the maximum of ionization calculated by some *a priori* models (e.g. the International Reference Ionosphere—IRI—2016 model^56,57^ and NeQuick model^58,59^—Fig. 6). We finally selected the NeQuick model because the computational time is faster then IRI, giving a great advantage for the real-time aproach. Anyway, we highlight that the two models are fully consistent. NeQuick model is based on a single input parameter—the Effective Ionisation Level (Az)—determined using three coefficients broadcast in the Galileo navigation message^60^; this resulted, for the day of the Chile event, in a maximum electron density height of 290 km.

The TEC time series were analysed with VARION during the time window from 21:45:00 to 23:59:59 GNSS time. The longer period (> 35–40 min) component of the TEC variation was removed using an 8$^{th}$ order polynomial fit in order to highlight the AGW$_{epi}$^29^. We highlight that—in our knowledge—this is the first time that the AGW$_{epi}$ is detected in a near real-time scenario.

The elevation cut-off angle of 20$^\circ$ was used in order to remove the noisier observations as usually suggested in the literature^2^. The bottom panels of Fig. 2 (the right panel is a zoom in of the green rectangle of the left one) give a general vision of sTEC time series variations for all satellites and all stations, referred to the respective IPP positions 8 min after the earthquake, and sorted by the IPP corresponding distances (positive and negative distances represent respectively the IPP placed north and south of the epicenter from the epicenter at this epoch). It is important to highlight that overall 11 GPS satellites (G02, G05, G06, G12, G13, G14, G15, G20, G24, G25, G29) observed from the 118 GPS permanent stations showing the much higher spatial coverage of the ionospheric observations (bottom panels of Fig. 2) compared to the GNSS ground motion observation only (upper panel of Fig. 2). The right panel of Fig. 4 shows the area analysed in which the coloured tracks represent the sTEC variations at the SIPs (same positions of the corresponding IPPs projected on the Earth ellipsoid) from the earthquake time (22:54:49 GPS time) to 23:59:59 GPS time. The TEC perturbation computed in real-time by VARION and showed in the right panel of Fig. 4 is characterized by a favorable coverage and is consistent with the source extent, proving the potential advantages to use real-time sTEC observations for tsunami genesis estimation. Additionally, sTEC observations appear to correlate well with the vertical ground motion and the related kinetic energy showing a strong perturbation mainly located at the North of the epicenter. Video S1 in Supplementary Material shows the TEC perturbation appearing about 9.5 min after the earthquake and moving northward.

The remarkable spatial density of ionospheric TEC observation by GNSS compared to ground motion (shown in Figs. 2 and 4) is simply related to fact that ground information is calculated only at the GNSS station location, instead the TEC measurements are located at the IPP between the GNSS station and the satellites. Consequently, at each epoch, every GNSS station has one single measurement of ground motion but several sTEC measurements: nominally, one for each satellite in view. The actual multiplication of GNSS systems—i.e., GPS (US), GLONASS (Russia), Galileo (EU), BeiDou (China)—is opening ambitious perspectives for really dense coverage of ionospheric monitoring to support conventional ground motion observation by seismometers.

**Figure 7:**
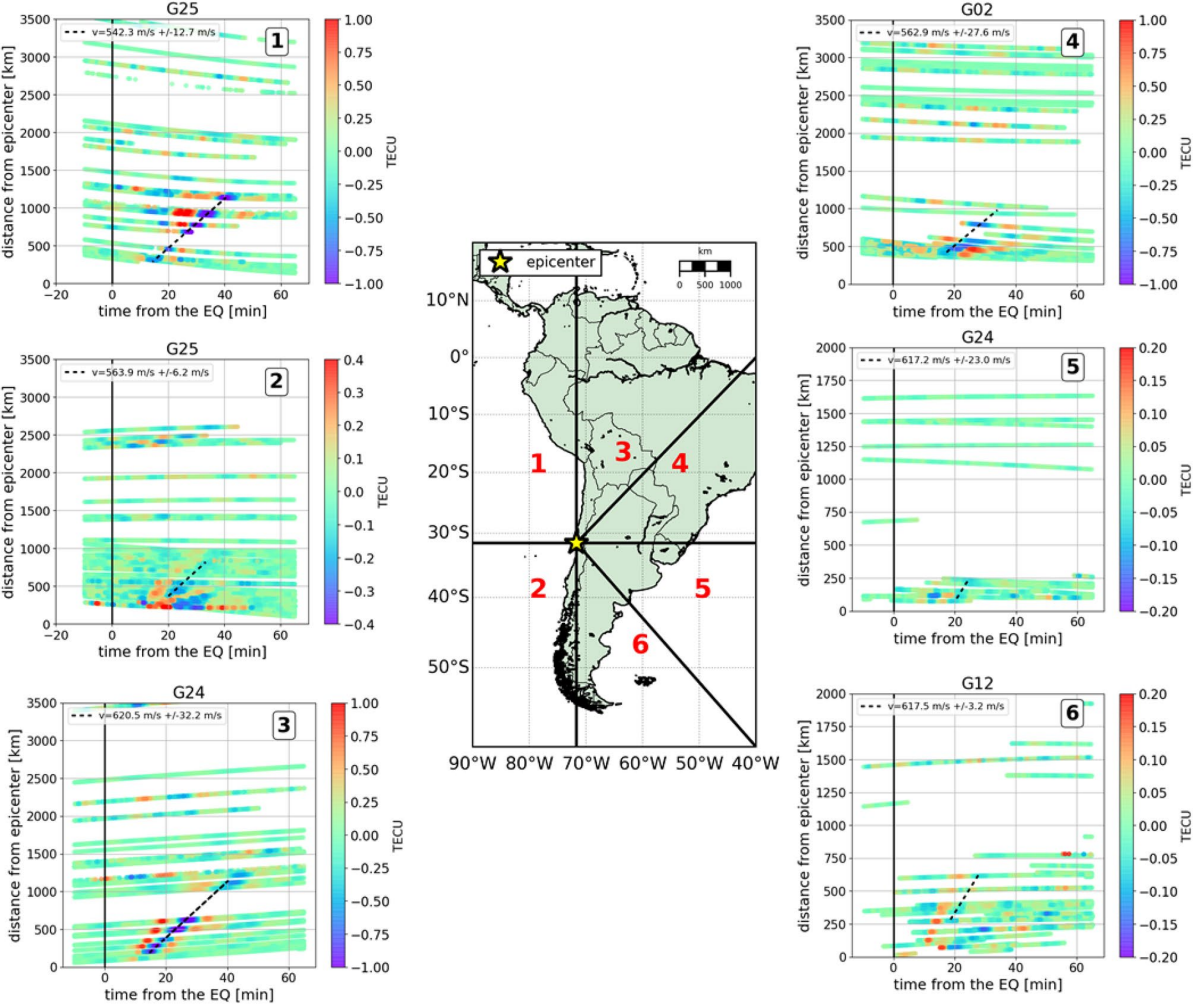
Map indicating the division in six regions of the studied area making the epicenter the area center. The two western sectors (1 and 2) are mainly placed over the Pacific Ocean, while the four eastern sectors (3, 4, 5 and 6) are mainly placed over land. Hodochron plots for the 6 regions identified computed for a time interval of one hour and 15 min (from 22:45:00 to 23:59:59 GPS time) and for some satellites in view (cut-off angle of 20$^\circ$) from all the 118 GPS stations. The number in the upper right box denotes the region to which the hodochrones belong. The slope of the straight line fitted, considering a linear least-squares regression for corresponding sTEC minima for different satellites, represent the AGW_epi_ horizontal propagation velocity.

Figure 7 summarizes more than one hour (from 22:45:00 to 23:59:59 GPS time) of GNSS sTEC observations around the epicenter (hodochrones) and clearly shows the propagation of the AGW$_{epi}$ all around from the epicenter at the speed of sound. The propagation speed of the AGW$_{epi}$ was estimated by linear least-squares regression and the horizontal disturbance propagation velocities are estimated with their standard deviations within the range of 620–640 m/s +/− 40 m/s (see Method for comparison with models). To better illustrate the correspondence between vertical ground shaking and ionospheric sTEC disturbances, the area was divided into six zones, four mainly on land and two mainly on the Pacific Ocean. The oblique straight lines, separating regions 3 and 4, and regions 5 and 6, have azimuths respectively equal to 45 and 135 degrees. We selected the satellites with the best observation geometry well highlighting the sTEC perturbation (other satellites are showed in the Fig. SM5). As expected, and coherently with the ground motion observations, the TEC perturbations are more evident in the north of the epicenter (region 1, 3 and 4). This effect is also partially induced by the magnetic field lines^18,61^.

## Conclusions

VADASE and VARION variometric algorithms were defined and implemented in the past years to estimate ground shaking, co-seismic displacements and ionosphere disturbances from GNSS data in real time. In this paper we combined the two algorithms in a single one so-called Total Variometric Approach (TVA); only GNSS data that are really available in real time were analized in a real-time scenario with TVA to retrieve the ground shaking, the energy associated to its Up component and the ionospheric signature due to the September 16, 2015 Mw 8.3 Illapel earthquake in Chile. We highlight that both ground-motion and TEC measurements reveal the north-south asymmetry related to the geometry of the rupture, and coherently with the plate tectonic local characteristic.

The proposed real-time Total Variometric Approach (TVA) provides us with both ground shaking and TEC perturbation in real time, on the basis of the same GNSS data stream. The TVA applied to the case of study of the Illapel earthquake (Mw 8.3, Chile) in 2015 using the data stream of 118 GNSS stations all around the epicenter allows to estimate significant ground shaking at only 30 s after the rupture as well as the consequent energy related to the shaking. Additionally, the anomalous TEC perturbation, estimated with the same GNSS data-stream, and related to the propagation of the AGW$_{epi}$, is detected at 9.5 min after the rupture and it also displays the north-south asymmetry previously shown by the GNSS ground-motion measurements. The high spatial resolution of the GNSS-TEC observations reveal the source extent, the potential advantages introduced by the ionospheric observations. We highlight that no classic techniques are today able to directly measure the source extent of the rupture during an earthquake.

Therefore, for the very first time, we have demonstrated to simultaneously monitor—using the Total Variometric Aproach (TVA) on the same real-time GNSS data-stream—the ground-motion and the ionospheric TEC perturbation to support traditional instrument (e.g., seismometers, accelerometers, buoys and tide gages) to improve the quick estimation of the source parameters and to estimate the tsunami genesis.

Considering the richness of the information coming from ionospheric sounding and the demonstrated possibility to detect the ionospheric perturbation related to the AGW$_{epi}$ in about 9.5 min, TVA can be already used for to support classic tsunami warning system. Indeed, following Manta *et al.*^16^ the ionospheric tsunami power index (ITPI) of the Illapel event in 2015 is 14.51, corresponding to the estimated water-volume in the order of 10$^4$ km$^3$ displaced during the tsunami genesis.

Consequently, the final goal of the paper is to underline the feasibility and reliability of TVA approach for tsunami detection. Indeed, the TVA main advantages is to leverage the information coming from the ionosphere to densify the GNSS observations and to make up for the lack of any other data. This makes TVA suitable for regions without high GNSS coverage (e.g., Sumatra and Pacific region) or a massive scientific infrastructure (e.g., supercomputers). Furthermore, TVA represents a low-cost tool, ready to be implemented on existing high-rate GNSS real-time networks and to push forward the installation of new dense GNSS real-time networks. Finally, TVA main goal is to represent an additional resource to classic methods, such as tsunami forecast^62^, earthquake early warning for tsunami alert^63^, tsunami inundation simulation and damage estimation^64,65^; in this context, the synergic use of all the available tools could really enhance the already existing tsunami warning systems.

## Methods

**Total Variometric Approach** The proposed real-time method is called Total Variometric Approach (TVA) and it is based on the joint use of VARION and VADASE algorithms. Both algorithms share the same variometric principle (time single-difference of proper combinations of phase observations) using a standalone GPS receiver and standard GNSS broadcast products (orbits and clock corrections) that are available in real-time; VADASE uses ionosphere-free combination and it estimates GNSS station velocities and displacements (thus earthquake ground shaking)^41,42^, while VARION uses geometry-free combination and it estimates sTEC variations^29^. VADASE and VARION can run in parallel but independently using the same GNSS data stream, so that TVA represents a comprehensive way to GNSS ground and ionosphere seismology, ready to be implemented on still existing high-rate GNSS permanent networks able to supply real-time data. Here data processing with both VADASE and VARION was performed off-line, even if in a real-time scenario: exclusively the information available in real time was used.

**VADASE** starting from the previous developed and published methodology^41,42^, the algorithm was improved with statistical test to define the significant shaking duration on the basis of the mean noise level of the velocity components, which have been recognized to be constant before and after the earthquake shaking generally with Gaussian distribution. East, North and Up velocities before and after the earthquake were evaluated to determine whether data are normally distributed. The result from D’Agostino–Pearson^66^ test shows that the data follows a Gaussian distribution as shown in Fig. SM6 of the Supplementary material. The incidence of non-normality is equal at most to 12.5%. For this reason, Levene test for equality of variances^67^ was chosen instead of the F-test applied in Fratarcangeli *et al.*^42^: Levene test is, indeed, less powerful, but, at the same time, less sensitive to departure from normality than F-test. The Levene test was performed on the horizontal velocity component (sum of East and North components).

Starting from the duration of significant shaking, we defined the overall coseismic displacements and the energy released by the earthquake. The series of the coseismic displacements, computed from the integration of VADASE velocity solutions, for the East, North and Up component, were de-trended as follows. First, we computed the spatial median of the displacements for the stations within 300 km of the epicenter as reported in Fratarcangeli *et al.*^42^. In our case, we computed two different medians—respectively for the northward and the southward stations—since they are impacted differently by the rupture. The spatial median was fitted robustly with a linear model (Huber regression model^68^) considering the interval of 60 s before the earthquake for the nearest stations (CMBA in the north and LVIL in the south). This straight line was extrapolated on all the interval of the coseismic displacements and then removed. Once completed the de-trending procedure, the overall coseismic displacements were computed using the strategy reported in Fratarcangeli *et al.*^42^. Based on the de-trending estimation, we also determined the uncertainty (sigmas) on the coseismic displacements using the model reported in Equation 1. More in detail, $\Delta _{Cr}$ represents the overall coseismic displacements estimation; a and b are respectively the slope and y-intercept of the robusted estimated linear model; $\sigma ^{2}_{a}$, $\sigma ^{2}_{b}$ and $\sigma _{ab}$ are the variances of the slope and y-intercept and the covariance between a and b respectively; $\Delta t$ is the significant shaking duration.

Regarding the energy produced by the Up component of ground shaking released by the earthquake, we computed the Power Spectral Density (PSD) to the ground shaking duration for the up component of the velocity at each site. The PSD was estimated using Welch method: an improvement of the standard periodogram spectrum estimation method, able to reduce the noise in the estimated power spectra in exchange for reducing the frequency resolution^69,70^. By integrating the PSD within a specific frequency range, the energy (in that frequency range) was calculated. This range is limited below by the specific cut-off frequency for the AGW$_{epi}$, as reported in the Introduction section. In this way, the normalized (for unit mass) kinetic energy for the Up component, related to the earthquake shaking, was computed for each site.

1 $$\begin{aligned} \sigma ^{2}_{\Delta _{Cr} } = \begin{vmatrix} \Delta t&1\\ \end{vmatrix} \begin{vmatrix} \sigma ^{2}_{a}&\sigma _{ab} \\ \sigma _{ab}&\sigma ^{2}_{b} \\ \end{vmatrix} \begin{vmatrix} \Delta t \\ 1\\ \end{vmatrix} \end{aligned}$$

**Atmospheric velocity of AGW**$_{epi}$ The estimated velocity of the AGW$_{epi}$ by the analysis of Fig. 7 were also validated with the US Naval Research Laboratory Mass-Spectrometer Incoherent-Scatter (NRL MSISE-00) and IRI-2016 models. The NRL MSISE-00 is an empirical, global model of the Earth’s atmosphere from ground to thermospheric heights whose outputs are temperatures and densities of the atmosphere components^71^. Hence, the temperature T profile as a function of the height in the earthquake day was computed. Then the sound velocity, $c_s=\sqrt{\frac{\gamma T R}{m_1}}$, where R (approximately 8.3145 J mol$^{-1}$ K$^{-1}$), is the molar gas constant, $\gamma$ is the adiabatic index (1.6667 for monatomic gases and 1.4 for diatomic gases), and $m_1$ is the molar mass of the gases, was evaluated. The left panel of Fig. 6 shows the sound velocity profile for the earthquake day: a velocity of about 910 m/s is estimated at an height of 290 km (F2 layer height, where the electron density is maximum). This sound velocity derived from model is coherent with the estimated sTEC perturbations velocity range, since hodochrones represent only the horizontal propagation velocity component of the AGW$_{epi}$ at the level of the F2 maximum, not the full velocity along the propagating k-vector.

## Supplementary Information

Supplementary Information.

Supplementary Information.
