# Supplementary material for: GNSS total variometric approach: first demonstration of a tool for real-time tsunami genesis estimation
Source: Sci Rep. 2021 Feb 4;11:3114. doi: 10.1038/s41598-021-82532-6 (PMC7862306; doi:10.1038/s41598-021-82532-6)
Supplement: Supplementary file 1 — Supplementary Information. [file 41598_2021_82532_MOESM1_ESM.pdf]

GNSS Total Variometric Approach: First Demonstration of a Tool for Real-Time Tsunami Genesis Estimation

Michela Ravanelli[1,*], Giovanni Occhipinti[2,3], Giorgio Savastano[4,5], Attila Komjathy[4], Esayas B. Shume[4,+], and Mattia Crespi[1]

[1]Sapienza University of Rome, via Eudossiana 18, Rome, 00184 Italy
[2]Université de Paris, Institut de Physique du Globe de Paris, CNRS, F-75005 Paris, France.
[3]Institut Universitaire de France, France.
[4]Jet Propulsion Laboratory,4800 Oak Grove Dr, Pasadena, CA 91109, USA
[5]now at Spire Global, Inc., 33 Rue Sainte-Zithe, 2763 Luxembourg, Luxembourg
[+]now at California Institute of Technology, 1200 E California Blvd, Pasadena, CA 91125, USA
[*]michela.ravanelli@uniroma1.it

ABSTRACT

Supplementary Information

Supplementary Info

The data coming from accelerometers were also analysed to estimate the normalized kinetic energy released by the earthquake. The dataset was provided by the *Centro Sismológico Nacional, Universidad de Chile* (CSN) website. The data reports the ground accelerations of East, North and Up components for 44 stations placed at about 300 km from the epicenter as it is possible to see in the Figure SM1. Data sampling rate was 100 or 200 samples per second; on the other hand, the accelerometer data cover a shorter time span (about 5 minutes of data) with respect to the GPS data (the entire day of the earthquake). In order to apply the same procedure of the GPS data, the measured accelerations were integrated for obtaining velocities for the East, North and Up components. At the same time, the accelerometers take more time to reach again their undisturbed situation, since they are more sensible instruments with respect to the GPS. For this reason, the test statistic to detect the significant shaking duration was not applied. On this point, in order to compute the normalized kinetic energy, a time interval starting from the earthquake time to the end of data (about 5 minutes after the earthquake time) was adopted. For the remaining part, we applied the same procedure described in the Methods section. Analogously to GPS data, the different steps to the energy determination are depicted in Figure SM2 .

Figure SM3 is geographic representation of the earthquake ground shaking computed by the accelerometers: the size of each station marker is proportional to the energy released by the earthquake in that point. Similarly to GPS permanent station, also in this case, it is possible to affirm that generally north-placed stations are characterized by a higher ground shaking energy. Nevertheless, in this case, it is less evident than in the analysed GPS observations. Indeed, there are some stations at north in which this difference is not present. We may interpret this aspect as due to different features of the local site conditions or station characteristics.

Figure SM4 depicts the the coseismic displacements series as computed by integrating the VADASE velocity solutions. It is also possible to highlight the different spatial median computed for north and south stations.

The supplementary video S1 shows the sTEC variations at the SIPs from the earthquake time to 50 minutes after, at intervals of 30 seconds. The Video demonstrates once again that the first detection epoch, when a clear the AGW_{epi} apperars is around 9.5 minutes after the earthquake. Furthermore, it is also possible to notice that the highest ionospheric disturbances is always concentrated in the northern areas of the epicenter.

Figure SM5 shows hodochrons plot for other satellites for each of the six region in which the study area was divided. The slope of the straight line fitted, considering a linear least-squares regression for corresponding sTEC minima for different satellites, represent the AGW_{epi} horizontal propagation velocity.

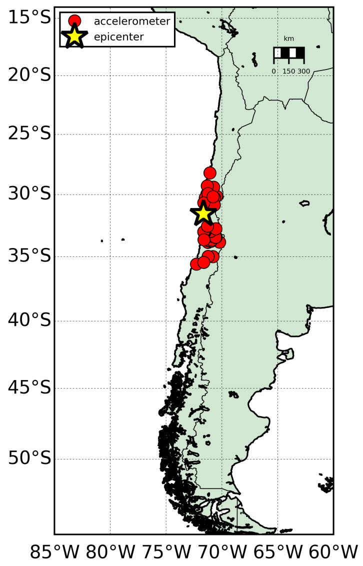

Figure 1. Map representing the acceleromter dataset, provided by CSN.

Figure SM6 shows the statistical distribution of the North component of velocity for CMBA GPS station before and after the earthquake. It is possible to see that data follow a normal distribution: the D'Agostino-Person is, hence, verified.

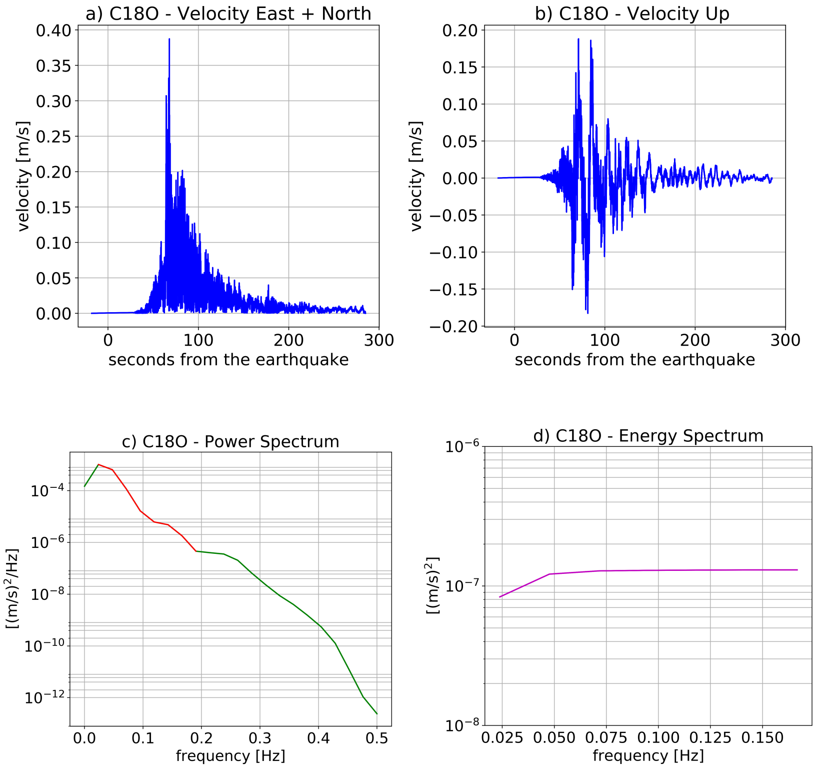

Figure 2. **(a, b)** Velocity time series for C18O accelerometer station placed north of the epicenter. Specifically, **(a)** and **(b)** show the single horizontal component and the Up component of the velocity respectively. **(c)** The PSD for C18O station. In red the chosen frequency range (3.3-200 mHz) for integrating is highlighted. **(d)** The energy spectrum for C18O station. It is remarkable that north placed stations are characterized by higher energy values than south placed ones.

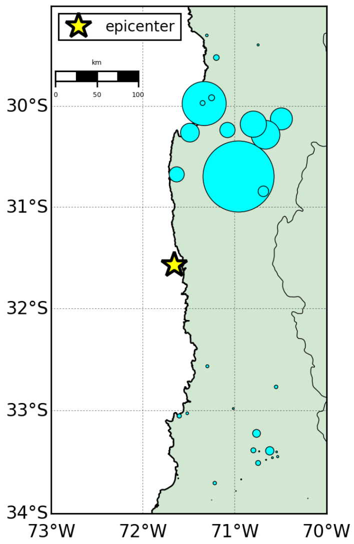

Figure 3. The map is a geographic representation of the earthquake ground shaking computed by the accelerometers: the dimension of the station markers is proportional to the energy associated to the Up component of the earthquake ground shaking for some stations near the epicenter. It is evident that generally north placed stations are characterized by a higher energy than south placed ones.

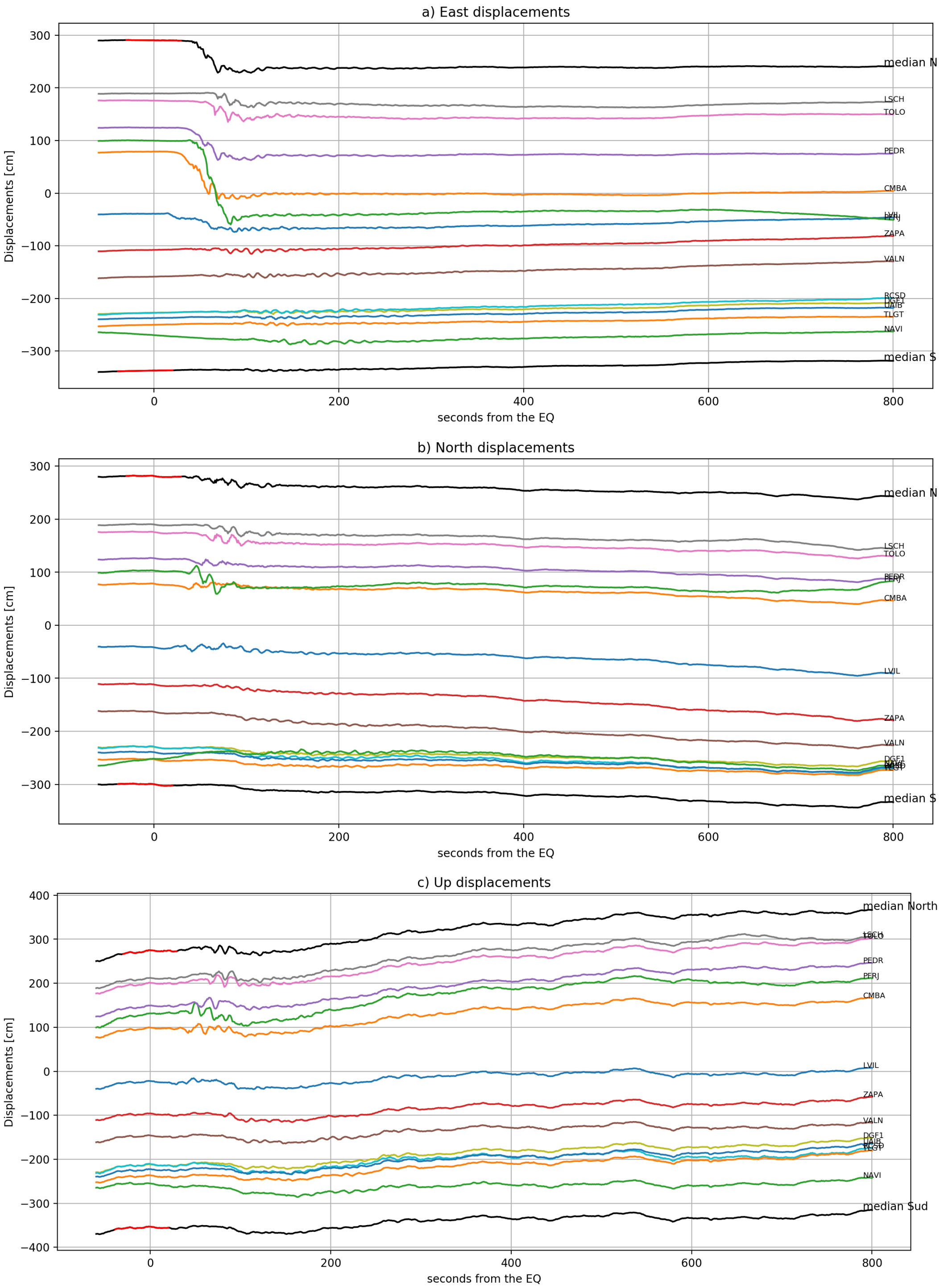

Figure 4. VADASE coseismic displacements for the East (a), North (b) and Up (c) component. The stations are ordered according to their distance from the epicenter (positive distances for north placed stations and negative distances for south placed stations). The spatial median computed for the north and south stations is depicted in black; the red part highlights the interval over which the robust linear fit was estimated.

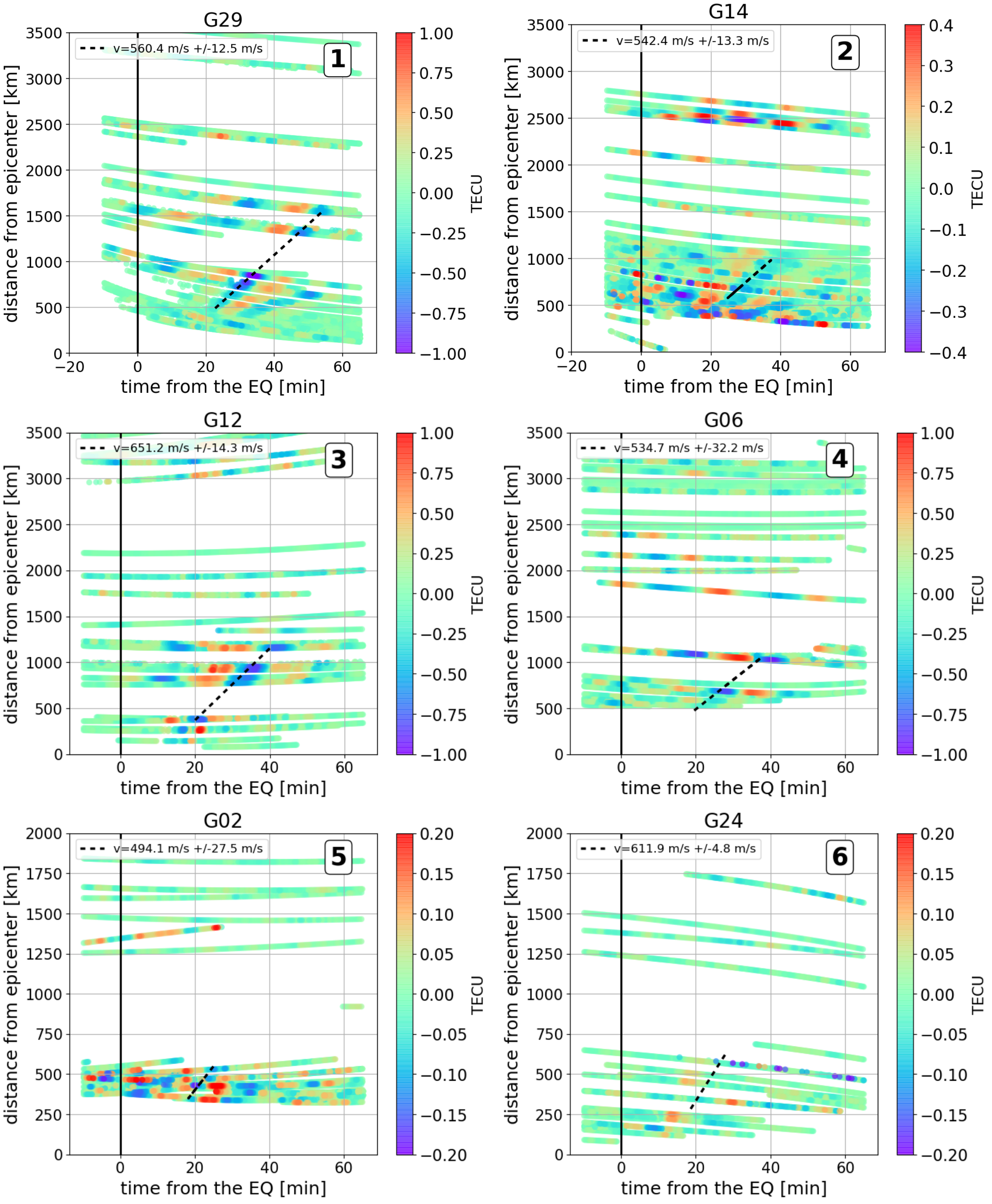

Figure 5. Hodochron plots for the 6 regions identified computed for a time interval of one hour and 15 minutes (from 22:45:00 to 23:59:59 GPS time) and for some satellites in view (cut-off angle of 20°) from all the 118 GPS stations. The number in the upper right box denotes the region to which the hodochrons belong. The slope of the straight line fitted, considering a linear least-squares regression for corresponding sTEC minima for different satellites, represent the AGW$_{epi}$ horizontal propagation velocity.

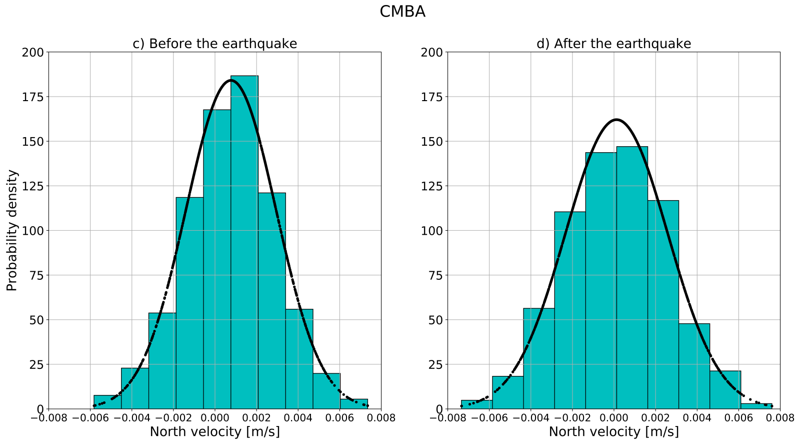

Figure 6. **(a, b)** The statistical distribution of North velocities before (left panel) and after (right panel) the earthquake time for station CMBA placed north of the epicenter. In both cases, all the normality tests are verified: data follow a Gaussian distribution.
